# KRASness and PIK3CAness in Patients with Advanced Colorectal Cancer: Outcome after Treatment with Early-Phase Trials with Targeted Pathway Inhibitors

**DOI:** 10.1371/journal.pone.0038033

**Published:** 2012-05-31

**Authors:** Ignacio Garrido-Laguna, David S. Hong, Filip Janku, Ly M. Nguyen, Gerald S. Falchook, Siqing Fu, Jenifer J. Wheler, Rajyalakshmi Luthra, Aung Naing, Xuemei Wang, Razelle Kurzrock

**Affiliations:** 1 Department of Investigational Cancer Therapeutics (Phase I Clinical Trials Program), The University of Texas MD Anderson Cancer Center, Houston, Texas, United States of America; 2 Department of Molecular Diagnostic Laboratory, The University of Texas MD Anderson Cancer Center, Houston, Texas, United States of America; 3 Department of Biostatistics, The University of Texas MD Anderson Cancer Center, Houston, Texas, United States of America; University de Minho, Portugal

## Abstract

**Purpose:**

To evaluate clinicopathologic and molecular features of patients with metastatic colorectal cancer (mCRC) and their outcomes in early-phase trials using pathway-targeting agents.

**Patients and Methods:**

We analyzed characteristics of 238 patients with mCRC referred to the phase 1 trials unit at MD Anderson Cancer Center. *KRAS*, *PIK3CA* and *BRAF* status were tested using PCR-based DNA sequencing.

**Results:**

Fifty-one percent of patients harbored *KRAS* mutations; 15% had *PIK3CA* mutations. In the multivariate regression model for clinical characteristics *KRAS* mutations were associated with an increased incidence of lung and bone metastases and decreased incidence of adrenal metastases; *PIK3CA* mutations were marginally correlated with mucinous tumors (p = 0.05). In the univariate analysis, *KRAS* and *PIK3CA* mutations were strongly associated. Advanced Duke's stage (p<0.0001) and *KRAS* mutations (p = 0.01) were the only significant independent predictors of poor survival (Cox proportional hazards model). Patients with *PIK3CA* mutations had a trend toward shorter progression-free survival when treated with anti-EGFR therapies (p = 0.07). Eighteen of 78 assessable patients (23%) treated with PI3K/Akt/mTOR axis inhibitors achieved stable disease [SD] ≥6 months or complete response/partial response (CR/PR), only one of whom were in the subgroup (N = 15) with *PIK3CA* mutations, perhaps because 10 of these 15 patients (67%) had coexisting *KRAS* mutations. No SD ≥6 months/CR/PR was observed in the 10 patients treated with mitogen-activating protein kinase (MAPK) pathway targeting drugs.

**Conclusions:**

*KRAS* and *PIK3CA* mutations frequently coexist in patients with colorectal cancer, and are associated with clinical characteristics and outcome. Overcoming resistance may require targeting both pathways.

## Introduction

There is increasing support for the concept that specific mutations predict the clinical manifestations and response to therapy of patients with cancer. In colorectal cancer, *RAS* mutations have received mounting scrutiny. In particular, activating mutations in *KRAS* drive resistance to anti-EGFR therapies in patients with metastatic disease. [1], [2] However, a subset of individuals with *KRAS* mutations (p.G13D) might derive benefit from anti-EGFR therapies. [3] The role of *PIK3CA* mutations in predicting resistance to anti-EGFR therapies has been debated, with initial studies reaching opposing conclusions. [4], [5] Recently, a multicenter retrospective study showed that *PIK3CA* mutations in exon 20 were involved in resistance to anti-EGFR therapies, whereas mutations in exon 9 were not. [6] Finally, our group demonstrated that activating mutations in *PIK3CA* may predict response to PI3K/Akt/mTOR inhibitors. [7]

Here, we review the clinical and molecular characteristics of patients with metastatic colorectal cancer (mCRC) who were referred to our clinical trials unit. The purpose of the study was to identify clinical characteristics associated with *KRAS* and *PIK3CA* mutations, and outcomes on early clinical trials of PI3K/Akt/mTOR and MAPK inhibitors.

## Results

### Patient Characteristics

Patient and tumor clinicopathologic characteristics for the 238 study patients are listed in Table 1. Fifty-four percent of patients were men. Seventy-one percent of patients were over the age of fifty. Most patients (69%) were Caucasians. The most common sites of metastases were liver, lymph nodes and lung, found in 83%, 75%, and 72% of patients, respectively. All 238 patients were tested for *KRAS* status. One-hundred-and-twenty-two patients (51%) had *KRAS* mutations. Of the 168 patients tested for *PIK3CA* mutations, 25 (15%) had a mutation. Of the 173 patients tested for *BRAF* mutations, 11 (6%) had a mutation.

**Table 1:** Patient characteristics.

VARIABLES	Overall, N = 238	*KRAS* wild-type N = 116	*KRAS* mutation N = 122	p-value
Gender				
Male	128 (54%)	63 (54.3%)	65 (53.3%)	0.90
Female	110 (46%)	53 (45.7%)	57 (46.7%)	
Age (years)				
≤50	68 (29%)	33 (28.5%)	35 (28.7%)	0.94
>50	170 (71%)	83 (71.5%)	87 (71.3%)	
Race				
White	165 (69%)	82 (70.7%)	83 (68.0%)	0.25
Hispanic	21 (9%)	13 (11.2%)	8 (6.6%)	
African-American	41 (17%)	15 (12.9%)	26 (21.3%)	
Other	11 (5%)	6 (5.2%)	5 (4.1%)	
Histology				
Non-mucinous	197(17%)	99 (85.3%)	98 (80.3%)	0.39
Mucinous	41 (83%)	17 (14.7%)	24 (19.7%)	
Sitê				
Ascending	56 (24%)	21 (18.6%)	35 (28.9%)	0.28
Transverse	15 (6%)	7 (6.2%)	8 (6.6%)	
Descending/sigmoid	95 (41%)	48 (42.5%)	47 (38.8%)	
Rectum	68 (29%)	37 (32.7%)	31 (25.6%)	
Dukes stage#				
A	1 (0.4%)	0 (0%)	1 (0.8%)	0.51
B	19 (8%)	10 (8.8%)	9 (7.4%)	
C	79 (33%)	42 (36.8%)	37 (30.3%)	
D	137 (58%)	62 (54.4%)	75 (61.5%)	
*BRAF* *				
wild-type	162 (94%)	83 (88.3%)	79 (100%)	**0.002**
mutated	11 (6%)	11 (11.7%)	0 (0%)	
*PIK3CA* **				
wild-type	143 (85%)	77 (91.7%)	66 (78.6%)	**0.03**
mutated	25 (15%)	7 (8.3%)	18 (21.4%)	
Adjuvant chemo				
None	17 (7%)	7 (13.5%)	10 (20.8%)	0.43
Yes	83 (93%)	45 (86.5%)	38 (79.2%)	
Anti-EGFR therapy				
No	109 (46%)	15 (12.9%)	94 (77.0%)	0.0001
Yes	129 (54%)	101 (87.1%)	28 (23.0%)	
Metastases				
Liver	198 (83%)	94 (81.0%)	104 (85.2%)	0.39
Lung	172 (72%)	76 (65.5%)	96 (78.7%)	**0.03**
Adrenal gland	31 (13%)	19 (16.4%)	12 (9.8%)	0.18
Brain	9 (4%)	4 (3.4%)	5 (4.1%)	1.00
Peritoneum	81 (34%)	36 (31.0%)	45 (36.9%)	0.41
Lymph nodes	179 (75%)	85 (73.3%)	94 (77.0%)	0.55
Ovarian	14 (6%)	8 (6.9%)	6 (4.9%)	0.59
Bone	35 (15%)	11 (9.5%)	24 (19.7%)	**0.03**

**^Site** at diagnosis was unknown for four patients,

# **Dukes stage** at diagnosis was unknown for two patients,

* **BRAF** status was known in 173 patients,

** **PIK3CA** status was known in 168 patients

### Frequency of *KRAS* Mutation Subtypes

Next, we assessed the incidence of different types of *KRAS* mutations. The location of *KRAS* mutations was available in 108 out of 122 patients. The most frequent *KRAS* mutation was p.G12D (31 patients [29%]), followed by p.G12V (23 patients [21%]), p.G12A (14 patients [13%]) and p.G13D (13 patients [12%]). Other mutations occurred at the following low incidences: p.G12C (11 patients [10%]), p.G12S (8 patients [7%]), p.Q61H (5 patients [5%]) and p.G12R, p.G13C and p.G12F, one patient each (1%).

### Evaluation of “KRASness”

#### Clinical Characteristics

Patients with *KRAS* mutations (N = 122) had a higher incidence of lung (79% [96/122] vs. 66% [76/116], p = 0.03) and bone metastases (20% [24/122] vs. 9% [11/116], p = 0.03) compared to those with wild-type *KRAS* (N = 116).

To further assess the association between *KRAS* mutations and different clinical features in patients with colorectal cancer, we fitted univariate and multivariate logistic regression models. The univariate models suggested that patients with *KRAS* mutations had a higher probability of having lung (p = 0.02) and bone metastases (p = 0.03) (Table 2). We fitted a multivariate model including those variables with univariate p-values <0.5 in the full model. After backward model selection, the multivariate model showed that increased lung and bone metastases and decreased adrenal metastases are significantly associated with *KRAS* mutations (Table 2)

**Table 2:** Univariate and multivariate logistic regression model for clinical characteristics associated with *KRAS* mutations in colorectal cancer.

Univariate regression model (N = 238)						
Variable	Coefficient	SE	P-value	Odds Ratio	95% Confidence Interval	
Age at diagnose >50 (vs. ≤50)	−0.01	0.29	0.97	0.99	0.56	1.74
Male (vs. female)	−0.04	0.26	0.87	0.96	0.58	1.60
Mucinous (vs. non-mucinous)	0.35	0.35	0.31	1.42	0.72	2.82
Dukes = a, b, c (vs. d)	−0.29	0.26	0.27	0.75	0.45	1.26
Liver metastases = yes (vs. no)	0.3	0.35	0.39	1.35	0.68	2.68
Lung metastases = yes (vs. no)	0.66	0.3	**0.02**	1.94	1.09	3.46
Adrenal = yes (vs. no)	−0.59	0.39	0.14	0.56	0.26	1.21
Brain metastases = yes (vs. no)	0.18	0.68	0.79	1.20	0.31	4.57
Peritoneal metastases = yes (vs. no)	0.26	0.27	0.34	1.30	0.76	2.23
Lymph node metastases = yes (vs. no)	0.2	0.3	0.50	1.22	0.68	2.21
Ovarian metastases = yes (vs. no)	−0.36	0.56	0.52	0.70	0.24	2.08
Bone metastases = yes (vs. no)	0.85	0.39	**0.03**	2.34	1.09	5.02

Multivariate Regression Model (N = 238)						
Intercept	−0.40	0.25	0.12	-	-	-
Lung mets = yes (vs. no)	0.61	0.31	0.05	1.85	1.01	3.36
Bone mets = yes (vs. no)	0.83	0.41	0.05	2.29	1.02	5.14
Adrenal = yes (vs. no)	−0.86	0.42	0.04	0.42	0.19	0.96

#### Molecular Characteristics

One-hundred-and-sixty-eight patients had testing for both *KRAS* and *PIK3CA* mutations. One-half of the patients tested (N = 84) had a *KRAS* mutation and one-half (N = 84) did not. Compared to *KRAS* wild-type patients, patients harboring *KRAS* mutations more frequently had *PIK3CA* mutations (21% [18/84] vs. 8% [7/84], p = 0.03). As previously reported, *BRAF* and *KRAS* mutations were mutually exclusive (173 patients tested for both) (Table 1). [6]

*PIK3CA* mutations were significantly associated with *KRAS* mutations in univariate models (p = 0.03). Of interest, when we introduced molecular features into the clinical multivariate model (i.e., *PIK3CA* status), the only variable associated with *KRAS* mutations was *PIK3CA* (data not shown).

### *KRAS* Mutations Were Associated With a Shorter OS

**Figure 1:**
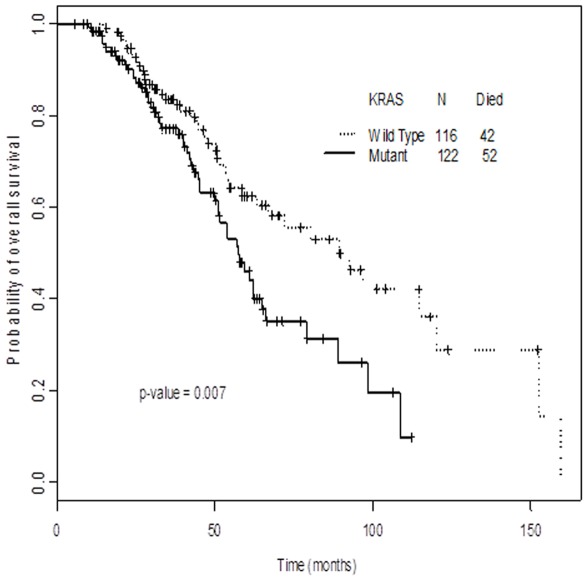
Kaplan-Meier plot of overall survival (OS) and *KRAS* status. Patients with *KRAS* wild-type mCRC had longer OS compared to *KRAS* mutant patients (tick marks represent patients still alive at time of last follow up).

We conducted a univariate analysis of survival for different patient characteristics, including age, gender, race, tumor type (mucinous vs. not), Duke's stage at diagnosis, site of primary tumor, *KRAS*, *BRAF* and *PIK3CA* mutations. In the multivariate analysis, Duke's stage at diagnosis and *KRAS* status were significant predictors of survival (*KRAS* HR 1.71, 95% CI 1.11–2.62) (Table 3). The median OS from time of diagnosis for patients with *KRAS* mutations was 57.5 months (95% CI: 50.0–64.8 months), whereas for *KRAS* wild-type patients, the median OS was 89.5 months (95% CI 63.5–120.1 months) (log-rank p = 0.007) (Figure 1).

**Table 3:** Univariate Analysis and Multivariate Cox Model for Overall Survival.

Univariate Cox proportional hazards model for overall survival					
Covariate	HR	95% CI	p-value	N death	N*
male (vs. female)	1.15	0.76–1.75	0.50	94	238
mucinous (vs. non-mucinous)	1.59	0.96–2.64	**0.07**	94	238
Dukes = a, b, c (vs. d)	0.38	0.25–0.60	**<0.0001**	93	236
*BRAF* = yes (vs. no)	1.28	0.46–3.55	0.63	64	173
*PIK3CA* = yes (vs. no)	1.15	0.61–2.17	0.67	63	168
*KRAS* = yes (vs. no)	1.77	1.16–2.70	**0.01**	94	238

Multivariate Cox proportional hazards model for overall survival (n = 236; death = 93)				
Dukes = a, b, c (vs. d)	0.39	0.25–0.60	<0.0001	
*KRAS* = yes (vs. no)	1.71	1.11–2.62	0.01	

We also assessed whether different types of *KRAS* mutations were associated with survival. We found no differences in OS across different types of *KRAS* mutations. For example, the median OS for patients with a codon 12 mutation was 57.5 months (95% CI 50–62.2 months) and the median OS for patients with a codon 13 mutation was 56.8 months (95% CI 28.8- not estimable) (log-rank p-value = 0.52). We further assessed OS, as defined by the specific type of amino acid involved in the different mutations. For this analysis we kept only subgroups with at least 10 observations (Table S1). There was no difference in OS. However, the number of patients in each subtype group was small, ranging from 11 to 31, precluding definitive conclusions.

### Evaluation of “PIK3CAness”

#### Clinical Features

To assess the association between *PIK3CA* mutations and different clinical features in patients with colorectal cancer, we tried to fit univariate and multiple logistic regression models. The univariate model suggested that patients with *PIK3CA* mutations more frequently had mucinous tumors (p = 0.04). They also had a trend to have less frequent liver metastases compared to patients with *PIK3CA* wild-type tumors (OR 0.40, p = 0.07) (Table 4). In the multivariate analysis, mucinous tumors were marginally significant (p-value = 0.05), with patients having mucinous tumors more frequently having *PIK3CA* mutations (OR 2.61; 95% CI 0.99–6.87). In addition, age showed a week trend (p-value = 0.15), with older patients less frequently having *PIK3CA* mutations (OR 0.52; 95% CI 0.22–1.26). As mentioned previously, *PIK3CA* status was not an independent variable predicting survival in multivariate analysis.

**Table 4:** Univariate and multivariate logistic regression model for clinical characteristics associated with *PIK3CA* mutations in colorectal cancer.

Univariate regression model (N = 168)						
variable	Coefficient	SE	P-value	Odds Ratio	95% Confidence Interval	
Age at diagnose >50 (vs. ≤50)	−0.70	0.44	0.11	0.49	0.21	1.18
male (vs. female)	0.54	0.46	0.24	1.72	0.70	4.25
mucinous (vs. non-mucinous)	1.01	0.49	**0.04**	2.75	1.05	7.14
Dukes = a, b, c (vs. d)	0.57	0.44	0.19	1.77	0.75	4.15
Liver metastases = yes (vs. no)	−0.93	0.51	**0.07**	0.39	0.15	1.07
lung metastases = yes (vs. no)	−0.19	0.47	0.68	0.83	0.33	2.06
Adrenal = yes (vs. no)	0.41	0.61	0.50	1.51	0.46	4.96
Brain metastases = yes (vs. no)	1.11	0.89	0.22	3.03	0.52	17.46
Peritoneal metastases = yes (vs. no)	0.47	0.44	0.28	1.60	0.68	3.81
LN metastases = yes (vs. no)	0.90	0.64	0.16	2.46	0.70	8.73
Ovarian metastases = yes (vs. no)	−0.79	1.06	0.46	0.45	0.06	3.66
Bone metastases = yes (vs. no)	0.10	0.59	0.86	1.11	0.35	3.55

Multivariate regression model (N = 168)						
Intercept	−1.54	0.38	<.0001	-	-	-
mucinous (vs. non-mucinous)	0.96	0.49	**0.05**	2.61	0.99	6.87
Age at Dx >50 (vs. ≤50)	−0.65	0.45	0.15	0.52	0.22	1.26

#### Molecular Characteristics

168 (71%) patients were tested for *PIK3CA* mutations. Of the 143 patients with wild-type *PIK3CA*, 66 (46%) had *KRAS* mutations. Of the 25 (15%) patients with *PIK3CA* mutations, 18 (72%) had *KRAS* mutations (p = 0.03) (Table 1). Of the 147 patients tested for both *PIK3CA* and *BRAF*, one patient (0.7%) had both mutations. A recent report showed that *PIK3CA* mutations in exon 20 were involved in resistance to anti-EGFR therapies, whereas mutations in exon 9 were not. [6] Further, although *KRAS* mutations are believed to predict resistance to EGFR inhibitor treatment, a subset of individuals with *KRAS* p.G13D mutations may still derive benefit from anti-EGFR therapies. [3] We, therefore, analyzed coexistent *PIK3CA* mutations (exon 9 and 20) in patients with *KRAS* mutations. We found that patients with *KRAS* p.G13D mutations had the lowest incidence of *PIK3CA* mutations (10%) compared to patients with *KRAS* p.G12A (36% had *PI3KCA* mutations), p.G12C (33%), p.G12D (22%) and p.G12V (12%). Moreover, none of the nine patients with *KRAS* p.G13D mutations had a *PIK3CA* mutation in exon 20, whereas *PIK3CA* mutations in exon 20 were found in all other types of *KRAS* mutations (Table S2). However, the small numbers of patients precludes drawing any statistically significant conclusions.

#### PFS on anti-EGFR treatment

We analyzed PFS in patients treated with anti-EGFR therapies (cetuximab or panitumumab). Patients with *KRAS* mutant mCRC treated with anti-EGFR therapies (N = 24) had shorter PFS compared to patients with wild-type *KRAS* (N = 98) (15 vs. 22 weeks, p = 0.01). Within the group of patients tested for *PIK3CA* mutations who received anti-EGFR therapies, patients with *PIK3CA* mutations (N = 9) had a trend toward a shorter PFS than *PIK3CA* wild-type patients (N = 82) (17 vs. 22 weeks, p = 0.07) (Figure S1).

### Response to PI3K/Akt/mTOR Inhibitors or MAP Kinase Pathway Inhibitors: impact of coexistence of *PIK3CA* and *KRAS* mutations

**Figure 2:**
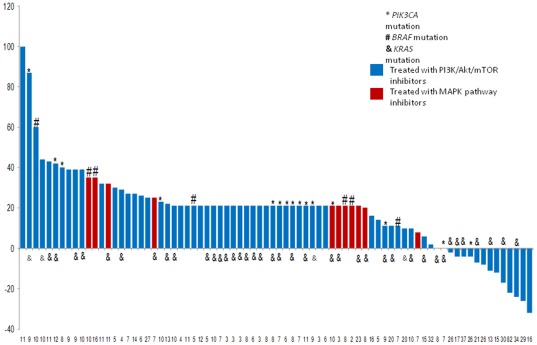
Waterfall plot with the responses by RECIST in the 78 assessable patients with mCRC that were treated with PI3K/AKT/mTOR inhibitors.

Eighty patients were treated on phase 1 protocols containing PI3K/Akt/mTOR inhibitors (NCT00454090, NCT00554268, NCT00610493, NCT00687622, NCT00726583, NCT00731263, NCT00756847, NCT00761644, NCT00770731, NCT00880321, NCT00920257, NCT00940381, NCT00972686, NCT01054313, NCT01072175, NCT01087554, NCT01087983, NCT01138085, NCT01155453, NCT01263145) (www.clinicaltrials.gov). Seventy-eight patients were assessable for response by RECIST (Figure 2). Of these 78 patients, 43 had a *KRAS* mutation in their tumors and 35 were *KRAS* wild-type. Of the 43 patients with *KRAS*-mutant disease, 9 (21%) attained SD ≥6 months/CR/PR; of the 33 patients with *KRAS* wild-type, 9 (27%) had SD ≥6 months/CR/PR (Fisher's exact test p = 0.59).

Fifteen patients with *PIK3CA* mutations were treated with PI3K/Akt/mTOR inhibitors, and one (7%) had SD ≥6 months/CR/PR; 10 of those patients (67%) had a concomitant *KRAS* mutation and none had a *BRAF* mutation. Sixty-three patients with wild-type or unknown *PIK3CA* status were treated with PI3K/Akt/mTOR inhibitors, and 17 (27%) had SD ≥6 months/CR/PR (Fisher's exact test for SD ≥6 months/CR/PR in *PIK3CA* mutant vs. wild-type p = 0.17); 33 of these patients (52%) had a *KRAS* mutation.

A total of 10 patients were treated with MAPK pathway inhibitors (generally BRAF or MEK inhibitors). Of those patients, four had *BRAF* and five had *KRAS* mutations. None had *PIK3CA* mutations. None of those patients had SD ≥6 months/CR/PR (figure 2).

## Discussion

In 238 patients with colorectal cancer referred to our clinical trials unit, we found that 122(51%) had *KRAS* mutations. The most frequent *KRAS* mutation subtypes were p.G12D (31 patients [25%]), followed by p.G12V (23 patients [19%]), p.G12A (14 patients [11%]) and p.G13D (13 patients [11%]). This incidence of *KRAS* subtypes was consistent with that reported in the Catalogue of Somatic Mutations in Cancer (COSMIC) database (www.sanger.ac.uk/genetics/CGP/cosmic/accessed on June 17^th^, 2011). In the multivariate analysis of clinical characteristics, *KRAS* mutations were associated with increased incidence of lung and bone metastases (p = 0.05 for each variable) and decreased incidence of adrenal metastases (p = 0.04) compared to *KRAS* wild-type patients. Previous studies found a higher probability of lung metastases in patients with mCRC with *KRAS* mutations. [8], [9] At the molecular level, patients harboring *KRAS* mutations more frequently had concomitant *PIK3CA* mutations compared to individuals with wild-type *KRAS* (21% vs. 8%, [p = 0.03]). Further, 72% of patients with *PIK3CA* mutations also had a *KRAS* mutation while only 46% of patients with wild-type *PIK3CA* had a *KRAS* mutation (p = 0.03). *PIK3CA* mutations were significantly associated with *KRAS* mutations in both univariate and multivariate models.

The median OS from time of diagnosis for patients with *KRAS* mutations was shorter than for those with wild-type *KRAS* (57.5 vs 89.5 months; p = 0.007) (Figure 1). Furthermore, in multivariate analysis, Duke's stage at diagnosis and *KRAS* status were significant independent predictors of survival. *BRAF* mutations have recently been found to be prognostic for shorter OS in patients with mCRC. [10], [11] However, the role of *KRAS* status as a predictor of OS is controversial. A previous retrospective study including 3,439 patients in a multivariate survival analysis found that only p.G12V mutations were predictive of poor survival. Interestingly, the incidence of p.G12V mutations in that study was considerably lower than the one reported in the COSMIC database (8% vs. 23%). A recent study showed increased OS in patients with *KRAS* wild-type mCRC treated with best supportive care compared to patients with *KRAS*-mutant mCRC. [1] However, after progressing on supportive care, patients with *KRAS* wild-type were allowed to cross over to panitumumab; therefore, treatment effect cannot be completely ruled out. It is unclear why different studies show conflicting data regarding the prognostic effect of *KRAS*, though it is conceivable that this is related to therapy given and/or other selection factors.

In univariate analysis, patients with *PIK3CA* mutations more frequently had mucinous tumors (p = 0.04), and tended to less frequently have liver metastases compared to patients with *PIK3CA* wild-type tumors. Only the association with mucinous tumor was (marginally) significant in the multivariate analysis (p = 0.05). *PIK3CA* status was not an independent variable predicting survival in multivariate analysis. At the molecular level, 72% of patients with *PIK3CA* mutations had coexisting *KRAS* mutations. Of possible interest, patients with mutated *PIK3CA* had a trend to a shorter PFS when treated with anti-EGFR therapies than those with wild-type *PIK3CA* (median = 22 weeks vs. 17 weeks, p = 0.07). Other reports also suggest that patients with *PIK3CA* mutations are less sensitive to anti-EGFR therapies. [4] However, a larger multicenter analysis recently found that only *PIK3CA* mutations in exon 20 were associated with a worse outcome after treatment with cetuximab. [6] Although several lines of evidence indicate that patients with colorectal cancer and *KRAS* mutations do poorly with EGFR inhibitors, recent data suggests that those individuals with *KRAS* mutation p.G13D may benefit from EGFR inhibitors. [3] We, therefore, analyzed patients with p.G13D *KRAS* mutations for exon 20 *PIK3CA* mutations and found none, whereas these mutations were found in other *KRAS* mutant subtypes. However, the small number of patients assessed precludes definitive conclusions. It may be warranted to evaluate, in prospective studies, whether or not co-existence of exon 20 mutated *PIK3CA* in certain subtypes of *KRAS* mutant disease could contribute to EGFR inhibitor resistance.

Eighty patients were treated on protocols containing PI3K/AKT/mTOR inhibitors of whom 78 (98%) were assessable. 23% achieved SD ≥6 months/CR/PR. There was no significant difference in the rate of SD ≥6 months/CR/PR between those with wild-type or mutant *KRAS*. Of interest, only 1 patient with a *PIK3CA* mutation achieved SD ≥6 months/CR/PR when treated with a PI3K/Akt/mTOR inhibitor. Most of these patients (67%) had co-existing *KRAS* mutations, which may explain their resistance. Indeed, activation of the MAPK pathway has recently been proposed as a mechanism of resistance to PI3K inhibitors. [12] Even so, it may be of interest that among the 13 patients that had some kind of tumor reduction, 7 had *KRAS* mutation. The latter suggests that though the rates of SD ≥6 months/CR/PR were low, and patients with co-existing *KRAS* and *PIK3CA* mutations did not respond, the presence of a *KRAS* mutation alone is not an absolute indicator of complete resistance to PI3K/Akt/mTOR inhibitor-based therapy. We recently reported that coexistence of *PIK3CA* and *KRAS* mutations was not predictive of resistance to PI3K/Akt/mTOR in patients with ovarian cancer. [13] Certainly, the disease-type context plays a role and additional mutations not included in this analysis may impact the response to these therapies. A recent work has shown that activation of a complex network of feed-back loops may for instance explain resistance to BRAF inhibitors in patients with colorectal cancer with *BRAF* mutations. [14]

Unexpectedly, 27% of patients with wild-type or unknown *PIK3CA* status treated with a PI3K/Akt/mTOR inhibitor attained SD ≥6 months/CR/PR (p = 0.09). It is possible that a molecular aberration in the PI3K/Akt/mTOR pathway existed in these patients but was not recognized, and that such an aberration may not have the same propensity to co-exist with *KRAS* or *BRAF* mutations. Indeed, loss of PTEN expression, which often indicates a *PTEN* mutation, has been reported in 40% of patients with metastatic colorectal cancer. [15]

A total of 10 patients (nine of whom had *KRAS* or *BRAF* mutations and none of whom had *PIK3CA* mutations) were treated with MAPK pathway inhibitors (generally BRAF or MEK inhibitors). Although the numbers of patients are small, none achieved SD ≥6 months/CR/PR.

Our work has several limitations. First, this is a retrospective study in a single institution with a relatively small number of patients. Second, we could not validate response rate to anti-EGFR therapies as this information was not available for many of the patients who were treated in institutions other than MD Anderson prior to being referred to our unit; hence, the only available clinical outcome for these patients was PFS. Third, all mutational analyses could not be completed for all patients included in the study because of limited amounts of available tissue.

In conclusion, we show that *KRAS* and *PIK3CA* mutations are frequently associated in patients with colorectal cancer. In the context of early clinical trials, drugs targeting the PI3K/Akt/mTOR pathway had limited activity in these patients, even in the presence of *PIK3CA* mutations, possibly because of the frequent coexistence of activating mutations in the MAPK pathway.

## Methods

### Patients

We retrospectively reviewed the clinicopathologic characteristics and clinical outcomes of 238 consecutive patients with mCRC who were seen in the Phase 1 Clinic (Clinical Center for Targeted Therapy) at The University of Texas MD Anderson Cancer Center beginning in October 2008, and for whom *KRAS* status was known. Data were collected from transcribed notes and radiology reports in the electronic database of these patients. The sites of metastatic disease were collected from the last available radiology report for each patient. Pathology was reviewed by an MD Anderson pathologist in all cases. The MD Anderson Cancer Center Institutional Review Board has approved the study. Written consent was given by the patients for their information to be stored in the hospital database and used for research.

### *KRAS, BRAF, PIK3CA* Mutation Testing

Mutation testing was done in the Clinical Laboratory Improvement Amendment-certified Molecular Diagnostic Laboratory within the Division of Pathology and Laboratory Medicine at MD Anderson. DNA was extracted from micro-dissected paraffin-embedded tumor and analyzed by a polymerase chain reaction (PCR)-based DNA sequencing method to examine codons 12, 13 and 61 of the *KRAS* proto-oncogene. The sensitivity of detection of this assay is approximately 1 in 10 mutation-bearing cells in the microdissected area. Whenever possible, analysis was done for *PIK3CA* mutations in codons [c] 532–554 of exon 9 (helical domain) and c1011–1062 of exon 20 (kinase domain) and *BRAF* c595–600 mutations of exon 15 by pyrosequencing as previously described. [16]

### Statistical Methods

Statistical analysis was performed and validated by our statistician (XW). Patient characteristics are summarized using descriptive statistics. The association between *KRAS* or *PIK3CA* mutation status and patient characteristics was assessed using Fisher's exact test. Overall survival (OS) is defined as the time interval between date of diagnosis and death. Patients who were alive were censored at the last follow-up date. Progression-free survival (PFS) was defined as the time from treatment initiation to detection of progressive disease or death. Patients who did not progress while on treatment were censored at the last date of follow up. The probabilities of OS and PFS were estimated using the method of Kaplan and Meier and were compared among subgroups of patients using the log-rank test. [17], [18] Cox proportional hazards regression models were fit to assess the association between OS and patient characteristics and *KRAS* mutation status. [19] Univariate and multiple logistic regression models were fit to assess the association between *KRAS* and *PIK3CA* mutation and patient clinical characteristics. Initially, univariate logistic regression models were fit and variables with p-values less than 0.5 were included in the multiple logistic regression model. We then performed backward model selection and kept only those variables with p-values less than 0.05. All statistical analyses were conducted using SAS software (version9.2) and Splus (version 8.0).

## Supporting Information

Figure S1 **Kaplan-Meier plot of progression-free survival (PFS) and** ***PIK3CA*** **status on patients with mCRC treated with regimens including anti-EGFR therapies.** Patients with mCRC/*PIK3CA* mutant had a trend toward a shorter PFS compared to *PIK3CA* wild-type patients.
(TIF)

Table S1 **Median overall survival (OS) for each group of** ***KRAS*** **mutations.**
(DOCX)

Table S2 **Type of** ***PIK3CA*** **mutations found in patients with** ***KRAS*** mutations*.
(DOCX)
