# Supplementary figures and images for: KRASness and PIK3CAness in Patients with Advanced Colorectal Cancer: Outcome after Treatment with Early-Phase Trials with Targeted Pathway Inhibitors
Source: PLoS One. 2012 May 31;7(5):e38033. doi: 10.1371/journal.pone.0038033 (PMC3364990; doi:10.1371/journal.pone.0038033)

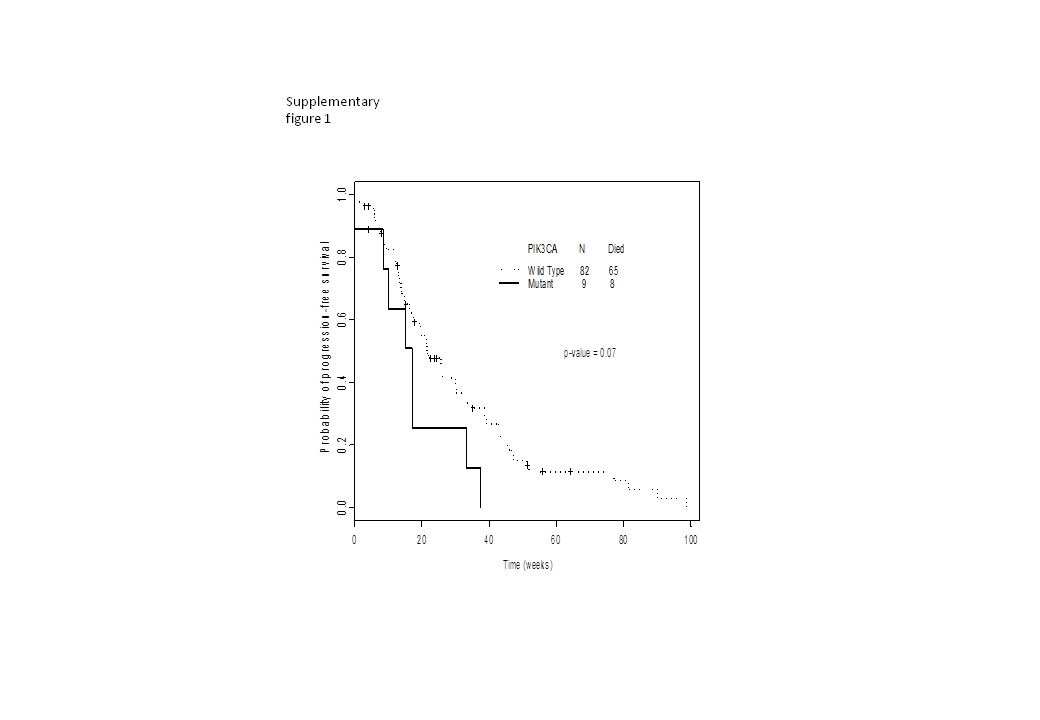

Supplement: Figure S1 — Kaplan-Meier plot of progression-free survival (PFS) and PIK3CA status on patients with mCRC treated with regimens including anti-EGFR therapies. Patients with mCRC/PIK3CA mutant had a trend toward a shorter PFS compared to PIK3CA wild-type patients. (TIF) [file pone.0038033.s001.tif]
