# Supplementary material for: KRASness and PIK3CAness in Patients with Advanced Colorectal Cancer: Outcome after Treatment with Early-Phase Trials with Targeted Pathway Inhibitors
Source: PLoS One. 2012 May 31;7(5):e38033. doi: 10.1371/journal.pone.0038033 (PMC3364990; doi:10.1371/journal.pone.0038033)
Supplement: Table S1 — Median overall survival (OS) for each group of KRAS mutations. (DOCX) [file pone.0038033.s002.docx]

**Supplementary Table 1.**

| **Group** | **N death/ Total** | **Median OS (months)** | **95% CI** |
| --- | --- | --- | --- |
| p.G12A | 5/14 | 44.8 | 22.2 – Not estimable |
| p.G12C | 8/11 | 50.0 | 14.3 – 59.3 |
| p.G12D | 14/31 | 61.0 | 38.4 – 108.6 |
| p.G12V | 9/23 | 62.1 | 45.4 – 98.3 |
| p.G13D | 6/13 | 56.8 | 28.8 – Not estimable |
