# Supplementary material for: KRASness and PIK3CAness in Patients with Advanced Colorectal Cancer: Outcome after Treatment with Early-Phase Trials with Targeted Pathway Inhibitors
Source: PLoS One. 2012 May 31;7(5):e38033. doi: 10.1371/journal.pone.0038033 (PMC3364990; doi:10.1371/journal.pone.0038033)
Supplement: Table S2 — Type of PIK3CA mutations found in patients with KRAS mutations*. (DOCX) [file pone.0038033.s003.docx]

**Supplementary Table 2.**

| ***KRAS* mutation** | ***PIK3CA* tested/ Total** | ***PIK3CA* mutation** | | **Incidence *PIK3CA*** | **Ratio Exon 20/9** |
| --- | --- | --- | --- | --- | --- |
|  |  | **Exon 9** | **Exon 20** |  |  |
| p.G12A | 11/14 | 2 | 2 | 36% | 50% |
| p.G12C | 6/11 | 1 | 1 | 33% | 50% |
| p.G12D | 23/31 | 4 | 1 | 22% | 20% |
| p.G12V | 15/23 | 1 | 1 | 13% | 50% |
| p.G13D | 10/13 | 1 | 0 | 10% | 0% |

*Only *KRAS* mutations observed in more than 10 patients were included
